# Serum leptin level and incidence of CKD: a longitudinal study of adult enrolled in the Korean genome and epidemiology study(KoGES)

**DOI:** 10.1186/s12882-022-02795-7

**Published:** 2022-05-26

**Authors:** Yon Chul Park, Solam Lee, Young-Sang Kim, Jae-Min Park, Kunhee Han, Hunju Lee, Kyung-Won Hong, Jong-Koo Kim, Eun Suk Cho, Tae-Ha Chung, Bom-Taeck Kim, Sang Baek Koh

**Affiliations:** 1 Department of Family Medicine, Wonju Severance Christian Hospital, Yonsei University, Wonju, Republic of Korea; 2 Department of Preventive Medicine, Yonsei University Wonju College of Medicine, Wonju, Republic of Korea; 3 Dept. of Family Medicine, CHA Bundang Medical Center, CHA University, Seongnam, Republic of Korea; 4 Department of Family Medicine, Gangnam Severance Hospital, Yonsei University, Seoul, Republic of Korea; 5 Department of Family Medicine, Seoul medical center, Seoul, Republic of Korea; 6 Theragen Bio Co., Ltd, Suwon, Republic of Korea; 7 Department of Family Practice and Community Health, Ajou University School of Medicine and Graduate School of Medicine, Suwon, Republic of Korea

**Keywords:** Leptin, Chronic kidney disease(CKD), Korean Genome and Epidemiology Study(KoGES)

## Abstract

**Background:**

Chronic kidney disease(CKD) is a major public health issue and is highly prevalent in the general population. Leptin is an adipose tissue-derived endocrine factor that has been associated with several metabolic factors involved in cardiovascular diseases. Several studies have investigated the association between leptin and renal diseases so far. But the results are conflicting between the studies. The objective of our study was to verify the direct association of serum leptin level with CKD development.

**Methods:**

This prospective cohort study included 2646 adult aged 40–70 without CKD in the Korean Genome and Epidemiology Study(KoGES) across South Korea from November 2005 to February 2012. The primary outcome was the development of CKD as defined by National Kidney Foundation Kidney Disease Outcomes Quality Initiative (KDOQI). Multivariate stepwise logistic regression analysis was done to assess the independent associations, for with the incident of CKD as the dependent variable, in tertiles of leptin values.

**Results:**

Among 1100 men and 1546 women with 2.8 mean years of follow-up, incidence of CKD was 18(1.63%) for men and 50(3.23%) for women. In the multivariate logistic regression models, individuals in the highest serum leptin tertile showed significant associations with risk of CKD after adjustment compared to the lowest tertiles in the population. The crude odds ratio for trend was 2.95(*p* = 0.004) for men. After adjusting for age, baseline eGFR variables showed correlation with statistical significance (OR for trend = 2.25, *p* = 0.037) for men. The same trends were also seen observed in all population and women also, but no statistical significance was found.

**Conclusions:**

Higher plasma leptin levels are associated with the incidence of CKD, independent of traditional factors such as age, baseline eGFR. Our results suggest that leptin may partly explain part of the reported association between obesity and kidney disease.

**Supplementary Information:**

The online version contains supplementary material available at 10.1186/s12882-022-02795-7.

## Background

Chronic kidney disease(CKD) is characterized by a progressive deterioration in renal function over a period of months or years accompanied by structural abnormalities of the kidney and other organs in the body, such as those involved in the skeletal and cardiovascular systems [1].

CKD is a major public health issue and is; highly prevalent in the general population. The National Health and Nutrition Examination Surveys showed that the prevalence of CKD increased from 10.0% in 1994–1998 to 13.1% in 1999–2004 [2]. CKD is extremely common among Asian populations. The prevalence of CKD in the adult Japanese population is 19.1% [3], and that in Beijing is 13.0% [4]. In the Korean population, the prevalence of CKD is 13.7% in those living in urban areas; according to these findings, 3.2 million urban Korean individuals aged 35 years or older may have CKD [5].

Reduced estimated glomerular filtration rate(eGFR) and excessive proteinuria are associated with increased risks of end stage renal disease (ESRD), CKD related cardiovascular diseases and other comorbidities [6]. CKD was defined as an eGFR of < 60 mL/min/1.73m2, consistent with the National Kidney Foundation Kidney Disease Outcomes Quality Initiative (KDOQI) guidelines for stage 3 chronic kidney disease [7] For the prevention of these detrimental consequences of CKD, it is essential to determine the highly predictable risk factors of CKD.

Leptin is an adipose tissue-derived endocrine factor that has been associated with several metabolic, inflammatory, and hemostatic factors involved in the development of hypertension and cardiovascular diseases [8]. Several studies have investigated the association between leptin and renal diseases, Previous experimental animal studies have suggested that higher leptin levels may cause hyperglycemia, elevations in blood pressure (mediated through increased sympathetic activity), and renal dysfunction [9]. In general population-based studies, higher plasma leptin levels were positively associated with CKD in a multi-ethnic, population-based sample of US adults [10].

Thus, to resolve this issue given the conflicting results between animal and human studies, we prospectively evaluated the relationship between baseline leptin levels and the new onset CKD events for 2.8 years in a Korean Genome and. Epidemiology Study(KoGES) cohort. This study aimed to verify the direct association of serum leptin level with CKD development.

## Methods

### Data Source and Study Approval

The Korean Genome and Epidemiology Study (KoGES) is a population-based prospective cohort in South Korea which is conducted in the Korean general population by the Korea National Institute of Health (KNIH). The participants have been followed-up every 2 years. The health check-up and measurements of biomarkers are carried out at each visit to identify risk factors for the development of chronic disease (e.g., hypertension, diabetes, osteoporosis, and cardiovascular disease), diet profile, lifestyle (e.g., alcohol intake, smoking, and exercise) and diverse environmental factors. The study was based on data Korean Genome and Epidemiology Study on the Atherosclerosis Risk of Rural Areas in the Korean General Population (KoGES-ARIRANG), part of the KoGES which included participants from 40–70 years of age who resided in the Wonju and Pyengchang areas of the Republic of Korea. In this study, 6,444 Korean adults aged 40–69 years were recruited from two Korean cities (Wonju and Pyeongchang), and were followed up biennially over a 7-year period [11–13]. The study was conducted in accordance with the Declaration of Helsinki. Informed written consent was obtained from all participants. Demographic information was collected at the baseline and follow-up examination using a standard questionnaire that was administered during face-to-face interviews. The study was approved by the institutional review board of Yonsei University Wonju College of Medicine (IRB No. 2020–02-0018).

### Study population

We used data from KoGES-ARIRANG to assess the incidence and risk factors of chronic kidney disease as per serum leptin level. Adults aged 40–70 years from the rural areas of Wonju and Pyeongchang were enrolled where demographic shifts are infrequent and high long-term follow up rates are expected. All study participants which did not done the first follow-up survey were first excluded. And individuals whose leptin levels were not evaluated at baseline, and whose GFR was less than 60 mL/min/1.73m^2^ also excluded.

### Endpoint definition

The endpoint was the development of CKD at the follow-up visit, defined based on the National Kidney Foundation KDOQI definition for chronic kidney disease (eGFR of < 60 mL/min/1.73m2). eGFR was calculated using the Modification of Diet in Renal Disease (MDRD) equation (eGFR = 186 × [serum Cr]-1.154 × [age]—0.203 × [0.742 if women]) which is known to be more accurate than other Equation [14].

### Statistical analysis

The data were expressed as means, medians, and frequencies. A t-test or Mann–Whitney U test was conducted to compare continuous variables. Meanwhile, the chi-square test was performed to compare categorical variables. All analyses were performed separately according to sex. Analysis of variance was performed to check the differences in the variables between the three groups. Multivariate logistic regression analysis was used to assess independent associations with incident CKD as the dependent variable in tertiles of leptin values. The potential confounder included age and baseline eGFR. No imputation was used for missing values as any subject with missing leptin and eGFR at baseline and follow-up were excluded during participant selection. The tertile cut-off points were 1.69 and 2.91 ng/mL for men (*n* = 1,039) and 6.08 and 9.89 ng/mL for women (*n* = 1,409). Results were expressed as odds ratios (ORs) along with their corresponding 95% confidence intervals (CIs). Age-adjusted ORs were first calculated (model 1), and the results were further adjusted for baseline eGFR (model 2). The results were further adjusted for age and baseline eGFR as continuous variables (model 3).

All analyses were performed using R version 3.6.2. *P* values of < 0.05 were considered significant.

## Results

### Cohort characteristics

**Fig.1:**
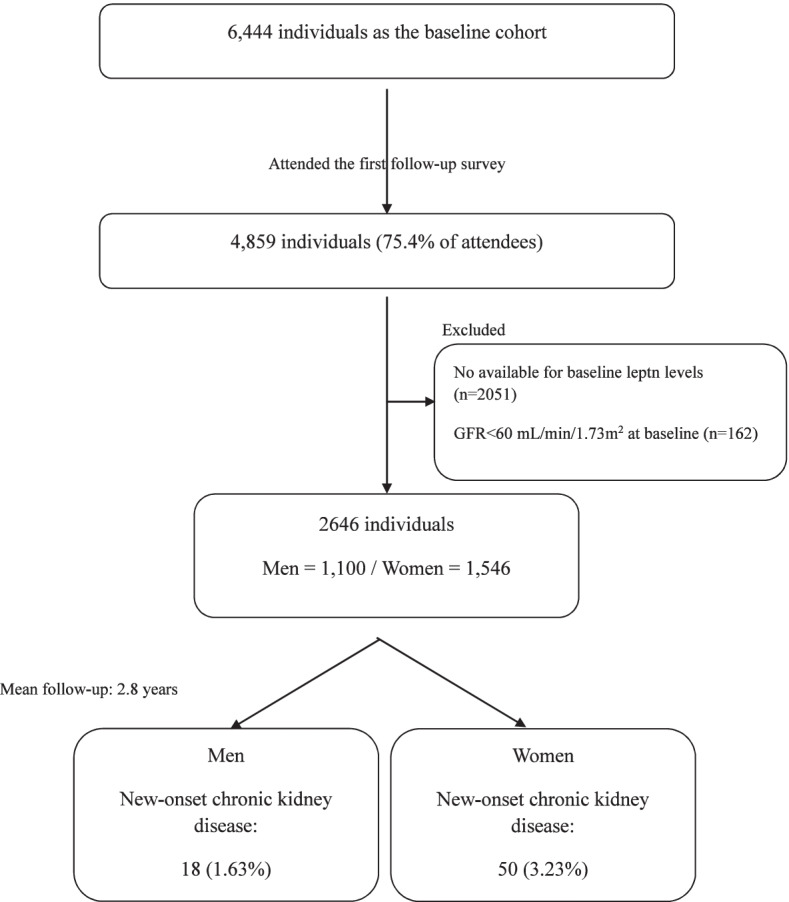
Study population

The baseline survey, carried out from November 2005 to February 2012, included 6,444 adults (2,563 men and 3,881 women) aged 40—70 years. All study participants were invited to the first follow-up survey (May 2008 to October 2017) and 4,859 (75.4%) attended. We excluded 2,051 individuals whose leptin levels were not evaluated at baseline, and 162 whose GFR was less than 60 mL/min/1.73m^2^. A total of 2,646 individuals were included in the final analysis (1100 men and 1546 women) (Fig. 1).

Table 1 shows the clinical and biochemical features of the study cohort. The mean age of the study population was 56.20 (standard deviation[SD]: 8.04) years for men and 54.14 (SD 8.18) years for women. The mean eGFR were 78.38 ± 10.81 mL/min/1.73m^2^ and 74.55 ± 8.86 mL/min/1.73m^2^ in men and women, respectively. The average BMI were 24.33 kg/m^2^ in men and 24.88 kg/m^2^ in women. The median leptin level in women (9.04 ng/mL, SD 2.04) was significantly higher than that in men (2.74 ng/mL, SD 5.64)(*P* < 0.001).

**Table 1:** Baseline characteristics of study population

		**Men**	**Women**	***P***
N	2646	1100	1546	< 0.001
Age, years	54.99 (8.18)	56.20 (8.04)	54.14 (8.18)	< 0.001
Hypertension, n (%)	550 (20.79)	213 (19.39)	337 (21.79)	0.135
Diabetes mellitus, n (%)	230 (8.70)	118 (10.75)	112 (7.22)	0.002
Dyslipidemia, n (%)	147 (5.54)	64 (5.79)	83 (5.36)	0.642
Myocardial infarction, n (%)	46 (1.73)	15 (1.38)	31 (1.99)	0.228
Cerebrovascular accident, n (%)	40 (1.50)	22 (2.020)	17 (1.13)	0.077
Current smoking, n (%)	463 (17.50)	441 (40.01)	22 (1.43)	< 0.001
Regular exercise, n (%)	720 (27.20)	272 (24.70)	448 (28.98)	0.014
Body mass index, kg/m^2^	24.65 (3.14)	24.33 (2.95)	24.88 (3.25)	< 0.001
Waist circumference, cm	83.67 (8.62)	86.40 (7.72)	81.72 (8.70)	< 0.001
Systolic BP, mmHg	127.20 (17.55)	129.01 (17.14)	125.91 (17.74)	< 0.001
Diastolic BP, mmHg	83.00 (11.79)	84.73 (11.34)	81.76 (11.95)	< 0.001
Fasting glucose, mg/dL	95.29 (19.92)	98.83 (24.05)	92.76 (17.83)	< 0.001
HbA1c	5.61 (0.80)	5.65 (0.85)	5.58 (0.76)	0.022
HDL-cholesterol, mg/dL	46.91 (11.09)	45.54 (11.59)	44.89 (10.62)	< 0.001
LDL-cholesterol, mg/dL	118.67 (33.07)	113.93 (32.35)	122.05 (33.17)	< 0.001
Triglycerides, mg/dL	147.94 (98.06)	167.00 (115.88)	134.39 (80.43)	< 0.001
HOMA-IR, units	1.98 (1.60)	1.97 (2.10)	1.99 (1.13)	0.782
hs-CRP, mg/L	2.07 (4.95)	2.40 (6.02)	1.82 (3.98)	0.006
eGFR, mL/min/1.73m^2^	76.14 (9.90)	78.38 (10.81)	74.55 (8.86)	< 0.001
Leptin, ng/L	6.42 (5.47)	2.74 (2.04)	9.04 (5.64)	< 0.001
BP, blood pressure; HDL, high density lipoprotein; LDL, low density lipoprotein; TG, triglyceride; HOMA-IR, Homeostasis Model Assessment of Insulin Resistance; hs-CRP, high sensitivity-C reactive protein; eGFR, estimated glomerular filtration rate				

The patients’ characteristics were divided in to three groups based on tertiles of leptin values; since leptin values differ according to sex, analysis were performed separately. (Table 2) For all populations, age, hypertension history, smoking history, BMI, fasting glucose levels, waist circumference, LDL-cholesterol levels, and Homeostasis Model Assessment of Insulin Resistance (HOMA-IR), baseline eGFR were different among the groups. In the male population, significant differences were observed in history of diabetes mellitus, history of dyslipidemia, systolic and diastolic blood pressure history of diabetes mellitus, history of hypertension, HbA1c and triglyceride levels, in addition to the aforementioned variables. In the female population, significant differences were observed in history of dyslipidemia, BMI, waist circumference, systolic and diastolic blood pressure, LDL-cholesterol levels, triglyceride levels, HOMA-IR, and hs-CRP level.

**Table 2:** Correlation between serum leptin level group and other components

	**Serum leptin level**			
	**Tertile 1**	**Tertile 2**	**Tertile 3**	***P***
All				
N (%)	872	873	901	
Age, years	56.53 (8.09)	54.36 (8.28)	54.13 (7.98)	< 0.001
Hypertension, n (%)	137 (15.7)	168 (19.3)	245 (27.18)	< 0.001
Diabetes mellitus, n (%)	75 (8.6)	89 (10.18)	66 (7.36)	0.350
Dyslipidemia, n (%)	40 (4.53)	50 (5.73)	57 (6.34)	0.100
Myocardial infarction, n (%)	9 (1.05)	17 (1.99)	19 (2.15)	0.078
Cerebrovascular accident, n (%)	13 (1.51)	18 (2.11)	8 (0.91)	0.293
Current smoking (%)	334 (38.25)	110 (12.65)	19 (2.12)	< 0.005
Regular exercise (%)	211 (24.25)	254.48 (29.15)	254 (28.17)	0.067
Body mass index, kg/m^2^	23.07 (2.60)	24.33 92.69)	26.49 (3.08)	< 0.001
Waist circumference, cm	82.48 (7.74)	82.88 (9.21)	85.57 (8.52)	< 0.001
Systolic BP, mmHg	126.89 (17.05)	126.91 (17.59)	127.77 (18.00)	0.295
Diastolic BP, mmHg	83.23 (11.49)	82.89 (11.78)	82.87 (12.09)	0.530
Fasting glucose, mg/dL	95.96 (21.02)	96.47 (22.37)	93.49 (15.76)	0.008
HbA1c	5.56 (0.80)	5.64 (0.93)	5.62 (0.67)	0.218
HDL-cholesterol, mg/dL	47.14 (11.79)	45.98 (11.03)	47.60 (10.39)	0.365
LDL-cholesterol, mg/dL	111.15 (31.86)	117.22 (31.63)	127.37 (33.61)	< 0.001
Triglycerides, mg/dL	149.12 (114.04)	145.38 (95.17)	149.29 (83.00)	0.964
HOMA-IR, units	1.59 (1.16)	2.05 (2.14)	2.29 (1.25)	< 0.001
hs-CRP, mg/L	2.12 (6.09)	2.02 (4.29)	2.06 (4.26)	0.825
Baseline eGFR, mL/min/1.73m^2^	78.57 (10.21)	75.87 (9.91)	74.05 (9.04)	< 0.001
Follow-up eGFR, mL/min/1.73m^2^	82.71 (11.72)	80.18 (11.45)	77.73 (10.45)	< 0.001
Leptin, ng/L	1.82 (0.69)	4.96 (1.18)	12.29 (5.40)	< 0.001
Male				
N (%)	362	363	375	
Age, years	57.20 (8.18)	55.79 (7.97)	55.62 (7.91)	0.008
Hypertension, n (%)	40 (11.08)	73 (20.11)	101 (26.83)	< 0.001
Diabetes mellitus, n (%)	15 (4.16)	48 (13.13)	56 (14.91)	< 0.001
Dyslipidemia, n (%)	13 (3.6)	17 (4.75)	34 (8.94)	0.002
Myocardial infarction, n (%)	3 (0.83)	4 (1.12)	8 (2.17)	0.121
Cerebrovascular accident, n (%)	3 (0.83)	9 (2.51)	10 (2.71)	0.072
Current smoking (%)	176 (48.75)	142 (39.05)	122 (32.53)	< 0.001
Regular exercise (%)	76 (21.11)	99 (27.22)	97 (25.74)	0.151
Body mass index, kg/m^2^	22.11 (2.19)	24.33 (2.21)	26.47 (2.63)	< 0.001
Waist circumference, cm	80.65 (6.40)	86.37 (5.92)	91.98 (6.19)	< 0.001
Systolic BP, mmHg	125.96 (16.15)	129.07 (17.15)	131.89 (17.58)	< 0.001
Diastolic BP, mmHg	82.86 (10.86)	84.55 (11.67)	86.70 (11.19)	< 0.001
Fasting glucose, mg/dL	94.17 (20.63)	99.82 (22.85)	102.37 (21.86)	< 0.001
HbA1c	5.51 (0.84)	5.65 (0.83)	5.79 (0.87)	< 0.001
HDL-cholesterol, mg/dL	48.58 (12.38)	45.02 (10.35)	43.12 (11.33)	< 0.001
LDL-cholesterol, mg/dL	107.58 (30.48)	114.75 (33.29)	119.25 (32.23)	< 0.001
Triglycerides, mg/dL	130.49 (93.56)	177.23 (134.73)	192.33 (106.79)	< 0.001
HOMA-IR, units	1.41 (1.11)	1.80 (1.32)	2.67 (3.04)	< 0.001
hs-CRP, mg/L	2.27 (7.78)	2.17 (4.81)	2.75 (4.99)	0.280
Baseline eGFR, mL/min/1.73m^2^	80.58 (10.66)	78.04 (9.93)	76.57 (11.42)	< 0.001
Follow-up eGFR, mL/min/1.73m^2^	85.25 (11.50)	82.03 (11.04)	81.18 (12.72)	< 0.001
Leptin, ng/L	1.15 (0.32)	2.20 (0.34)	4.79 (2.26)	< 0.001
Female				
N (%)	510	510	526	
Age, years	54.43 (8.49)	53.97 (8.06)	54.01 (7.98)	0.416
Hypertension, n (%)	71 (14)	120 (23.45)	145 (27.61)	< 0.001
Diabetes mellitus, n (%)	30 (5.88)	47 (9.22)	35 (6.56)	0.698
Dyslipidemia, n (%)	18 (3.6)	24 (4.75)	47 (8.94)	0.004
Myocardial infarction, n (%)	6 (1.22)	12 (2.4)	12 (2.32)	0.215
Cerebrovascular accident, n (%)	6 (1.22)	6 (1.2)	5 (0.97)	0.703
Current smoking (%)	9 (1.78)	5 (0.99)	8 (1.52)	0.736
Regular exercise (%)	145 (28.46)	156 (30.65)	147 (27.86)	0.825
Body mass index, kg/m^2^	22.50 (2.24)	24.80 (2.47)	27.25 (3.01)	< 0.001
Waist circumference, cm	76.52 (7.18)	81.44 (7.28)	87.04 (8.17)	< 0.001
Systolic BP, mmHg	122.96 (17.29)	125.25 (17.57)	129.40 (17.77)	< 0.001
Diastolic BP, mmHg	80.11 (11.92)	81.49 (11.87)	83.63 (11.81)	< 0.001
Fasting glucose, mg/dL	91.21 (18.06)	93.94 (20.41)	93.12 (14.55)	0.088
HbA1c	5.48 (0.83)	5.59 (0.77)	5.66 (0.68)	< 0.001
HDL-cholesterol, mg/dL	48.71 (10.94)	47.52 (10.70)	47.44 (10.17)	0.055
LDL-cholesterol, mg/dL	113.12 (28.76)	124.34 (33.71)	128.49 (34.78)	< 0.001
Triglycerides, mg/dL	110.03 (65.86)	142.41 (84.75)	150.23 (83.54)	< 0.001
HOMA-IR, units	1.58 (0.81)	1.96 (1.10)	2.41 (1.27)	< 0.001
hs-CRP, mg/L	1.51 (3.38)	1.83 (4.25)	2.12 (4.22)	0.017
Baseline eGFR, mL/min/1.73m^2^	75.41 (8.85)	74.40 (8.00)	73.85 (9.59)	0.005
Follow-up eGFR, mL/min/1.73m^2^	79.42 (11.28)	78.13 (9.63)	77.43 (10.80)	0.002
Leptin, ng/L	4.20 (1.22)	7.88 (1.08)	14.86 (5.74)	< 0.001
Values are expressed as mean (SD) or number (%) BP, blood pressure; HDL, high density lipoprotein; LDL, low density lipoprotein; TG, triglyceride; HOMA-IR, Homeostasis Model Assessment of Insulin Resistance; hs-CRP, high sensitivity-C reactive protein; eGFR, estimated glomerular filtration rate				

### Serum leptin and CKD

In the multivariate logistic regression models, individuals in the highest serum leptin tertile showed significant associations with CKD risk after adjustment compared with those in the lowest tertiles in the population. The odds ratio for trend was 1.15(*P* = 0.394) (Table 3). More pronounced tendency was observed in men. The odds ratio for trend was 3.19 after adjusting for age in men and statistical significance was noted (*P* = 0.002). Model 2 showed same tendency after adjusting eGFR. (OR for trend = 2.13, *P* > 0.049) Analyses after additionally adjusting for age and eGFR showed correlation with statistical significance between serum leptin and CKD risk (OR for trend = 2.25, *P* = 0.037). The same trends were also observed in women but no statistical significance was found.

**Table 3:** Odds ratios for new-onset chronic kidney disease according to baseline serum leptin level

**Serum leptin level**		**Odds ratio (95% CI)**			
	Tertile 1	Tertile 2	Tertile 3	Odds ratio for trend	*P* for trend
All (*n* = 2646)					
Serum leptin (ng/L)	< 3.09	3.09 – 7.23	≥ 7.23		
No. of new-onset CKD	14	26	28		
Crude	Reference	1.88 (0.98 – 3.63)	1.97 (1.03 – 3.76)	1.36 (1.00 – 1.83)	0.048
Model 1	Reference	2.25 (1.16 – 4.37)	2.44 (1.27 – 4.71)	1.50 (1.11 – 2.03)	0.009
Model 2	Reference	1.44 (0.74 – 2.82)	1.17 (0.60 – 2.28)	1.05 (0.77 – 1.43)	0.770
Model 3	Reference	1.62 (0.83 – 3.19)	1.41 (0.72 – 2.78)	1.15 (0.34 – 1.57)	0.394
Men (*n* = 1100)					
Serum leptin (ng/L)	< 1.68	1.68 – 2.87	≥ 2.87		
No. of new-onset CKD	2	3	13		
Crude	Reference	1.50 (0.25 – 9.03)	6.46 (1.45 – 28.83)	2.95 (1.41 – 6.18)	0.004
Model 1	Reference	1.76 (0.29 – 10.68)	7.91 (1.75 – 35.64)	3.19 (1.53 – 6.68)	0.037
Model 2	Reference	1.17 (0.19 – 7.21)	3.53 (0.76 – 16.30)	2.13 (1.00 – 4.53)	0.049
Model 3	Reference	1.14 (0.18 – 7.12)	3.81 (0.82 – 17.77)	2.25 (1.05 – 4.85)	0.037
Women (*n* = 1546)					
Serum leptin (ng/L)	< 6.07	6.07 – 9.77	≥ 9.77		
No. of new-onset CKD	17	13	20		
Crude	Reference	0.76 (0.37 – 1.58)	1.14 (0.59 – 2.21)	1.08 (0.76 – 1.52)	0.667
Model 1	Reference	0.80 (0.38- 1.68)	1.22 (0.63 – 2.37)	1.11 (0.79 – 1.57)	0.550
Model 2	Reference	0.70 (0.33 – 1.46)	0.91 (0.47 – 1.80)	0.96 (0.68 – 1.37)	0.829
Model 3	Reference	0.73 (0.34 – 1.53)	0.97 (0.49 – 1.91)	0.99 ()0.70 – 1.41)	0.967
Model 1: adjusted for age Model 2: adjusted for baseline eGFR Model 3: adjusted for age and baseline eGFR					

## Discussion

Our results showed that CKD recurred significantly more frequently in the high serum leptin group in men. The findings of some studies support this result. In a population-based study, higher plasma leptin levels were positively associated with CKD [9]. Leptin has shown positive correlations with high C-reactive protein levels [15], as well as with insulin resistance [16], which have been shown to be related to CKD [17, 18]. In the aspect of genetics, leptin gene expression is decreased in the adipose tissue of individuals with CKD, which may be a compensatory mechanism with regard to decreased clearance [19]. Moreover, infusion of recombinant leptin in rats triggered the development of focal glomerulosclerosis [20].

The study has several strengths. This is the first and only cohort study to establish the associations between leptin and CKD. As a prospective cohort study, the causal relationship between leptin and CKD can be analogize through this study.

Meanwhile, several limitations of our study should also be considered. First, although this is a prospective cohort study, the follow-up interval of the participants is diverse and the median f/u interval is only 2.8 years long. And the sample size was small, especially for the women, which are insufficient to perform multivariable logistic regression analysis of various variables. Second, the representativeness of the background population is limited because the study only included middle-aged and older Korean adults living in rural areas. Hence, our results may not be generalizable to other populations, such as urban residents, groups with different lifestyles or different incidence trends of CKD, and those with a younger age. Third, a single assessment of leptin and creatinine levels may be susceptible to short-term variations, which could bias the results toward null.

There are several hypotheses supporting this result. First, leptin activates the sympathetic nervous system and causes chronic elevations in blood pressure which leads to renal dysfunction [9]. Second, leptin induces natriuresis [21] which increases the arterial pressure to maintain the sodium and water balance [22]. Third, leptin, a cofactor in transforming growth factor-beta activation, promotes renal endothelial cell proliferation, and subsequently glomerulosclerosis [20, 23, 24].

Our results were only statistically significant in men.

Several leptin associated studies have also shown similar results according to sex difference. One study showed that a high leptin level independently predicted stroke in men but not in women [25]. Increased baseline leptin levels are associated with an increased risk of diabetes in men but not in women among Japanese Americans [26].

The role of sex based differences in the association between leptin and CKD still needs to be established; however, several suggestive pathophysiological clues have been reported.

First, leptin levels are higher in women than in men, suggesting an important biologic sex-specific difference due to lipid distribution [27].

Second, there is a physiological sex difference. In animal studies, the rate of leptin transport over the blood–brain barrier or during intracellular signaling cascades differs according to sex [28].

The role of leptin in mediating sympathetic nerve activation is well known. A previous study showed that infusions of leptin into rats increased the renal sympathetic nervous activity leading to CKD [20]. In the transgenic skinny mouse, which overexpresses leptin, systolic blood pressure and urinary catecholamine excretion were elevated [15]. It is possible that women may have lower responses or are less sensitive to the actions of leptin in the central sympathetic nervous system [29] These could be one of the reasons why the correlation was shown only in men.

## Conclusion

In conclusion, higher plasma leptin levels are associated with the incidence of CKD, independent of traditional factors such as age, BMI, systolic blood pressure, smoking, regular exercise, and levels of serum fasting glucose, HDL-cholesterol, and hs-CRP. Our results suggest that leptin may partly explain the reported association between obesity and kidney disease. However, future experimental studies are needed to confirm our findings which were only significant in men.

## Supplementary Information

**Additional file 1.** Supplement Table Odds ratios for new-onset chronic kidney disease according to baseline serum leptin level

**Additional file 2.** STROBE Statement—Checklist of items that should be included in reports of cohort studies

**Additional file 3.** Authorship form

## Data Availability

The data that support the findings of this study are available from the corresponding authors and the National Research Institute of Health of South Korea, but restrictions apply to the availability of these data, which were used under license for the current study and are not publicly available. Data are however available from the corresponding authors upon reasonable request and with permission of the National Research Institute of Health of South Korea. Further information is available at the KoGES website [http://www.nih.go.kr/contents.es?mid=a50401010100].

## Abbreviations

CKD: Chronic kidney disease
eGFR: Estimated glomerular filtration rate
ESRD: End stage renal disease
KDOQI: Kidney Disease Outcomes Quality Initiative
KoGES: Korean Genome and. Epidemiology Study
LDL: Low density lipoprotein
HDL: High density lipoprotein
TG: Triglycerides
hs-CRP: C-reactive protein
ORs: Odds ratios
Cis: Confidence intervals
BMI: Body mass index
SD: Standard deviation
HOMA-IR: Homeostasis Model Assessment of Insulin Resistance
