# Supplementary material for: Serum leptin level and incidence of CKD: a longitudinal study of adult enrolled in the Korean genome and epidemiology study(KoGES)
Source: BMC Nephrol. 2022 May 26;23:197. doi: 10.1186/s12882-022-02795-7 (PMC9137116; doi:10.1186/s12882-022-02795-7)
Supplement: Supplementary file 1 — Additional file 1. Supplement Table Odds ratios for new-onset chronic kidney disease according to baseline serum leptin level [file 12882_2022_2795_MOESM1_ESM.docx]

**Supplement Table** Odds ratios for new-onset chronic kidney disease according to baseline serum leptin level

| Serum leptin level |  | Odds ratio (95% CI) | | | |
| --- | --- | --- | --- | --- | --- |
|  | Tertile 1 | Tertile 2 | Tertile 3 | Odds ratio for trend | *P* for trend |
| All (n=2646) |  |  |  |  |  |
| Serum leptin (ng/L) | < 3.09 | 3.09 – 7.23 | ≥ 7.23 |  |  |
| No. of new-onset CKD | 14 | 26 | 28 |  |  |
| Crude | Reference | 1.88 (0.98 – 3.63) | 1.97 (1.03 – 3.76) | 1.36 (1.00 – 1.83) | 0.048 |
| Model 1 | Reference | 1.62 (0.78 – 3.35) | 1.91 (0.94 – 3.86) | 1.36 (0.97 – 1.90) | 0.077 |
| Model 2 | Reference | 1.26 (0.60 – 2.64) | 1.13 (0.55 – 2.33) | 1.04 (0.74 – 1.48) | 0.811 |
| Men (n=1100) |  |  |  |  |  |
| Serum leptin (ng/L) | < 1.68 | 1.68 – 2.87 | ≥ 2.87 |  |  |
| No. of new-onset CKD | 2 | 3 | 13 |  |  |
| Crude | Reference | 1.50 (0.25 – 9.03) | 6.46 (1.45 – 28.83) | 2.95 (1.41 – 6.18) | 0.004 |
| Model 1 | Reference | 1.01 (0.14 – 7.25) | 5.47 (1.19 – 25.11) | 2.86 (1.28- 6.38) | 0.010 |
| Model 2 | Reference | 0.80 (0.11 – 5.88) | 3.17 (0.67 – 15.03) | 2.13 (0.95 – 4.81) | 0.068 |
| Women (n=1546) |  |  |  |  |  |
| Serum leptin (ng/L) | < 6.07 | 6.07 – 9.77 | ≥ 9.77 |  |  |
| No. of new-onset CKD | 17 | 13 | 20 |  |  |
| Crude | Reference | 0.76 (0.37 – 1.58) | 1.14 (0.59 – 2.21) | 1.08 (0.76 – 1.52) | 0.667 |
| Model 1 | Reference | 0.76 (0.34 – 1.70) | 1.00 (0.47 – 2.10) | 1.00 (0.68 – 1.47) | 0.995 |
| Model 2 | Reference | 0.71 (0.31 – 1.61) | 0.78 (0.36 – 1.68) | 0.89 (0.60 – 1.31) | 0.550 |

Model 1: adjusted for HbA1c

Model 2: adjusted for HbA1c and baseline eGFR
