# Supplementary material for: Serum leptin level and incidence of CKD: a longitudinal study of adult enrolled in the Korean genome and epidemiology study(KoGES)
Source: BMC Nephrol. 2022 May 26;23:197. doi: 10.1186/s12882-022-02795-7 (PMC9137116; doi:10.1186/s12882-022-02795-7)
Supplement: Supplementary file 3 — Additional file 3. Authorship form [file 12882_2022_2795_MOESM3_ESM.pdf]

SPRINGER NATURE

Change of authorship request form - Journals (pre-acceptance)

Important information. Please read.

- This form should be used by authors to request any change in authorship (adding/deleting authors) including changes in corresponding authors. This form should not be used for name changes. Please fully complete all sections. Use black ink and block capitals and provide each author's full name with the given name first followed by the family name.

- By signing this declaration, all authors guarantee that the order of the authors are in accordance with their scientific contribution, if applicable as different conventions apply per discipline, and that only authors have been added who made a meaningful contribution to the work.

- Please note, in author collaborations where there is formal agreement for representing the collaboration, it is sufficient for the representative or legal guarantor (usually the corresponding author) to complete and sign the Authorship Change Form on behalf of all authors, **next to the added/removed author(s)**. **(Complete Section 3, followed by Section 6.)**
  In author collaborations where there is no formal agreement for representing the collaboration and **there are more than 10 authors**, one may sign for all, provided the signer appends correspondence that attests that each of the authors have agreed to the change **and the added/removed authors sign the form**. **(Complete Section 3, followed by Section 6.)**

- Please note, we cannot investigate or mediate any authorship disputes. If you are unable to obtain agreement from all authors (including those who you wish to be removed) you must refer the matter to your institution(s) for investigation. Please inform us if you need to do this.

- If you are not able to return a fully completed form within **30 days** of the date that it was sent to the author requesting the change, we may have to withdraw your manuscript. We cannot publish manuscripts where authorship has not been agreed by all authors (including those who have been removed).

- Incomplete forms will be rejected.

- Please return/upload this form, fully completed, to the Journals Editorial Office. The Journal and/or Publisher will consider the information you have provided to decide whether to approve the proposed change in authorship. We may decide to contact your institution for more information or undertake a further investigation, if appropriate, before making a final decision.

SPRINGER NATURE Springer Nature is one of the world's leading global research, educational and professional publishers, created in May 2015 through the combination of Nature Publishing Group, Palgrave Macmillan, Macmillan Education and Springer Science+Business Media.

SPRINGER NATURE

Change of authorship request form - Journals (pre-acceptance)

Section 1: Please provide the current title of manuscript

Manuscript ID no.: BNEP-D-20-00864R1

Title: Serum leptin level and incidence of CKD: A Longitudinal Study of Adult Enrolled in the Korean Genome and Epidemiology Study(KoGES)

Section 2: Please provide the previous authorship, in the order shown on the manuscript before the changes were introduced. Please indicate the corresponding author by adding (CA) behind the name.

	First name(s)	Family name	**ORCID** or SCOPUS id, if available
1st author	YONCHUL	PARK	0000-0002-1787-0884
2nd author	SOLAM	LEE	0000-0001-6458-9449
3rd author	YONG-SANG	KIM	0000-0002-7397-5410
4th author	JAE-MIN	PARK	0000-0001-8873-8832
5th author	KUNHEE	HAN	0000-0001-8307-7692
6th author	HUNJU	LEE	0000-0003-0089-0069
7th author	KYUNG-WON	HONG	0000-0002-3137-4609
8th author	JONG-KOO	KIM	0000-0003-2880-277X
9th author	EUN SUK	CHO	0000-0003-0282-2262
10th author	TAE-HA	CHUNG	0000-0001-5873-3352
11th author	BUM-TAEK **(CA)**	KIM **(CA)**	0000-0002-0395-0410
12th author	SANG BAEK **(CA)**	KO **(CA)**	0000-0001-5609-6521

SPRINGER NATURE Springer Nature is one of the world's leading global research, educational and professional publishers, created in May 2015 through the combination of Nature Publishing Group, Palgrave Macmillan, Macmillan Education and Springer Science+Business Media.

SPRINGER NATURE

Change of authorship request form - Journals (pre-acceptance)

Section 3: Please provide a justification for change. Please use this section to explain your reasons for changing the authorship of your manuscript, e.g. what necessitated the change in authorship? Please refer to the (journal) policy pages for more information about authorship. Please explain why omitted authors were not originally included and/or why authors were removed on the submitted manuscript.

The order of authors on the title page of the original paper was not changed from the first time. However, during the online submission process, two co-corresponding authors could not be set, so after the 1st author, corresponding author1, corresponding author2, the 2nd author, the 3rd author…….. were posted in the order. And another thing is that there was another typo mistake of Pf. Bom Taeck Kim which the name was first wrote as Bum Tack Kim. We are really sorry for the mistake. And Affiliated institute also have been updated.
We are attaching this form to ensure and All authors are aware of the order. Thank you

Section 4: Proposed new authorship. Please provide your new authorship list in the order you would like it to appear on the manuscript. Please indicate the corresponding author by adding (CA) behind the name. If the Corresponding Author has changed, please indicate the reason under section 3.

	First name(s)	Family name (this name will appear in full on the final publication and will be searchable in various abstract and indexing databases)	Affiliated institute	E-mail address
1st author	YONCHUL	PARK	Wonju Severance Christian hospital	iamyonchul@yonsei.ac.kr
2nd author	SOLAM	LEE	Yonsei Univ. Wonju College of Medicine	solam@yonsei.ac.kr
3rd author	YONG-SANG	KIM	CHA Bundang Medical Center	zeroup@cha.ac.kr
4th author	JAE-MIN	PARK	Gangnam Severance Hospital	milkcandy@yuhs.ac
5th author	KUNHEE	HAN	Seoul medical center	hankawow@naver.com
6th author	HUNJU	LEE	Yonsei Univ. Wonju College of Medicine	hjlee5371@gmail.com
7th author	KYUNG-WON	HONG	Theragen Bio Co., Ltd.	kyungwon.hong@theragenetex.com
8th author	JONG-KOO	KIM	Wonju Severance Christian hospital	kimjk214@yonsei.ac.kr
9th author	EUN SUK	CHO	Wonju Severance Christian hospital	joseph555@yonsei.ac.kr
10th author	TAE-HA	CHUNG	Wonju Severance Christian hospital	medeus115@yonsei.ac.kr
11th author	BOM-TAECK **(CA)**	KIM **(CA)**	Ajou University School of Medicine and Graduate School of Medicine	lovesong@ajou.ac.kr
12th author	SANG BAEK **(CA)**	KOH **(CA)**	Yonsei Univ. Wonju College of Medicine	kohhj@yonsei.ac.kr

---
SPRINGER NATURE Springer Nature is one of the world's leading global research, educational and professional publishers, created in May 2015 through the combination of Nature Publishing Group, Palgrave Macmillan, Macmillan Education and Springer Science+Business Media.

Change of authorship request form - Journals (pre-acceptance)

Section 5: Author contribution, Acknowledgement and Disclosures. Please use this section to provide a new disclosure statement and, if appropriate, acknowledge any contributors who have been removed as authors and ensure you state what contribution any new authors made (if applicable per the journal or book (series) policy). Please ensure these are updated in your manuscript - after approval of the change(s) - as our production department will not transfer the information in this form to your manuscript.

New acknowledgements:

Not applicable

New Disclosures (financial and non-financial interests, funding):

Not applicable

New Author Contributions statement (if applicable per the journal policy):

Not applicable

State 'Not applicable' if there are no new authors.

SPRINGER NATURE Springer Nature is one of the world's leading global research, educational and professional publishers, created in May 2015 through the combination of Nature Publishing Group, Palgrave Macmillan, Macmillan Education and Springer Science+Business Media.

SPRINGER NATURE

Change of authorship request form - Journals (pre-acceptance)

Section 6: Declaration of agreement. *All* authors, unchanged, new and removed *must* sign this declaration.
(NB: Please print the form, (docu)-sign and return/upload a scanned copy. Please note that signatures that have been inserted as an image file are acceptable as long as it is handwritten. Typed names in the signature box are unacceptable.) *** Please delete as appropriate. Delete all of the bold if you were on the original authorship list and are remaining as an author.**

	First name	Family name		Signature	Date
1st author	YONCHUL	PARK	I agree to the proposed new authorship shown in section 4 **/and the addition/removal*of my name to the authorship list** /and the proposed change in corresponding author		Oct. 10th, 2021
2nd author	SOLAM	LEE	I agree to the proposed new authorship shown in section 4 **/and the addition/removal*of my name to the authorship list** /and the proposed change in corresponding author		Oct. 10th, 2021
3rd author	YONG-SANG	KIM	I agree to the proposed new authorship shown in section 4 **/and the addition/removal*of my name to the authorship list** /and the proposed change in corresponding author		Oct. 10th, 2021
4th authors	JAE-MIN	PARK	I agree to the proposed new authorship shown in section 4 **/and the addition/removal*of my name to the authorship list** /and the proposed change in corresponding author		Oct. 10th, 2021
5th author	KUNHEE	HAN	I agree to the proposed new authorship shown in section 4 **/and the addition/removal*of my name to the authorship list** /and the proposed change in corresponding author		Oct. 10th, 2021
6th author	HUNJU	LEE	I agree to the proposed new authorship shown in section 4 **/and the addition/removal*of my name to the authorship list** /and the proposed change in corresponding author		Oct. 10th, 2021
7th author	KYUNG-WON	HONG	I agree to the proposed new authorship shown in section 4 **/and the addition/removal*of my name to the authorship list** /and the proposed change in corresponding author		Oct. 10th, 2021

SPRINGER NATURE Springer Nature is one of the world's leading global research, educational and professional publishers, created in May 2015 through the combination of Nature Publishing Group, Palgrave Macmillan, Macmillan Education and Springer Science+Business Media.

Change of authorship request form - Journals (pre-acceptance)

	First name	Family name		Signature	Date
8th author	JONG-KOO	KIM	I agree to the proposed new authorship shown in section 4 **/and the addition/removal*of my name to the authorship list** /and the proposed change in corresponding author		Oct. 10th , 2021
9th author	EUN SUK	CHO	I agree to the proposed new authorship shown in section 4 **/and the addition/removal*of my name to the authorship list** /and the proposed change in corresponding author		Oct. 10th , 2021
10th author	TAE-HA	CHUNG	I agree to the proposed new authorship shown in section 4 **/and the addition/removal*of my name to the authorship list** /and the proposed change in corresponding author		Oct. 10th , 2021
11th author	BUM-TAEK **(CA)**	KIM **(CA)**	I agree to the proposed new authorship shown in section 4 **/and the addition/removal*of my name to the authorship list** /and the proposed change in corresponding author		Oct. 10th , 2021
12th author	SANG BAEK **(CA)**	KOH **(CA)**	I agree to the proposed new authorship shown in section 4 **/and the addition/removal*of my name to the authorship list** /and the proposed change in corresponding author		Oct. 10th , 2021

In case of author collaborations with formal agreement:

	Name of consortium/consortia	First name	Family name		Signature	Date
Representative/legal guarantor				I agree to the proposed new authorship shown in section 4 **/and the addition/removal*of my name to the authorship list** /and the proposed change in corresponding author		

Both added/removed authors should complete the information in the first table under Section 6.

---- End of form ----

SPRINGER NATURE Springer Nature is one of the world's leading global research, educational and professional publishers, created in May 2015 through the combination of Nature Publishing Group, Palgrave Macmillan, Macmillan Education and Springer Science+Business Media.
